# Role and contribution of the nurse in caring for patients with palliative care needs: A scoping review

**DOI:** 10.1371/journal.pone.0307188

**Published:** 2024-08-23

**Authors:** Sue Moran, Maria E. Bailey, Owen Doody

**Affiliations:** 1 Milford Care Centre, Castletroy, Limerick, Ireland; 2 Board Member, PSPA Ireland, Dublin, Ireland; 3 Department of Nursing and Midwifery, Health Research Institute, University of Limerick, Limerick, Ireland; University of Technology Sydney, AUSTRALIA

## Abstract

**Background:**

The provision of high-quality palliative care is important to nursing practice. However, caring for palliative care patients and their families is challenging within a complex everchanging health environment. Nonetheless the caring, artistic role of the nurse is fundamental to the care of the patient and family. However, this role is currently being overshadowed by the technical and scientific elements of nursing.

**Methods:**

A scoping review was conducted utilising Arksey and O’Malley’s framework to identify the role and contribution of nurses in caring for patients with palliative care needs. An open time period search of eight electronic databases (MEDLINE, CINAHL, Academic Search Complete, PsycINFO, EMBASE, Web of Science, Scopus and Cochrane Library) was conducted on the 8^th^ of March 2023 and updated on the 30^th^ of April 2024. Screening was performed independently by two reviewers against eligibility criteria with meetings between authors to discuss included papers and form a consensus. Data was extracted relating to palliative care nursing, methodology, key findings, and recommendations. The analysed and summarised data was mapped onto Oldland et al seven domains framework: (a) medical/nursing and technical competence, (b) person centred care, (c) positive interpersonal behaviours, (d) clinical leadership and governance, (e) promotion of safety, (f) management of the environment, and (g) evidence-based practice.

**Results:**

Fifty-five papers met the criteria for this review which describes the role and contribution of nurses in caring for palliative patients across all domains of professional practice. The review found the leading areas of nurse contribution were person centred, interpersonal and nursing care aspects, with leadership, managing the environment, patient safety and evidence-based practice evident but scoring lower. The contribution of the nurse in palliative care supports a biopsychosocial-educational approach to addressing the physical, emotional and social needs of patients with palliative care needs and their families across the care continuum.

**Conclusion:**

Nurses in palliative care engage in a wide range of roles and responsibilities in caring for patients and their families with palliative care needs. However, there remains minimal evidence on the assessment, intervention, and evaluation strategies used by nurses to highlight the importance of their role in caring for patients and their families in this area. The findings of this review suggest that the artistic element of nursing care is being diluted and further research with a focus on evidencing the professional competence and artistic role of the nurse in the provision of palliative care is required. In addition, research is recommended that will highlight the impact of this care on patient and family care outcomes and experiences.

## Background

Palliative care describes a specific approach/philosophy to patient care and is defined by the WHO as “an approach that improves the quality of life of patients (adults and children) and their families who are facing problems associated with life threating illness, prevents and relieves suffering through the early identification, correct assessment and treatment of pain and other problems, whether physical, psychosocial or spiritual” [1].This definition is reflective of several iterations that were revised over the years to reflect current thinking, practice and person-centred approaches, holistic assessment, and accessibility for all. In line with advances in palliative care, palliative nursing and medicine has developed as specialist areas of practice. Their origins stem from the work and philosophy of Cicily Saunders (1918–2005) founder of the modern hospice movement who argued “to provide quality care at the end-of-life we need to bring scientific knowledge and artistic skills to the bedside, we need to combine the art and science of palliative care, we should learn not only how to free patients from pain and distress, but also how to be silent, how to listen, and how just to be there” (p:8) [2].

From a nursing perspective palliative nursing is described as: “a combination of knowledge, skills and compassion in equal measure, which is sensitive, hopeful, meaningful and dynamic, above all it is a way of thinking and an attitude of mind that should influence a nurse’s behaviour whenever they work with a dying person in whatever setting” (p.8) [3]. For the purposes of this paper, we have considered the provision of palliative nursing in the following settings: specialist palliative care and non-specialist settings in the hospital and community (practice nursing; district nursing and nursing homes). Evident within the literature, is that nursing values place patient care at the core of nursing and argue that recurring values of kindness, dignity, commitment, and competency are fundamental to palliative nursing care [4,5]. Nursing values are viewed as important, worthwhile, and worth striving for [6] and the unique role that nurses play in palliative care is evolving significantly as an “art” of nursing with nursing skills based on compassion, empathy and genuine kindness which are equated with equal measure to that of the science of nursing [7]. Nursing values are part of the nursing profession providing a framework which guides the nurse’s goals, behaviours, and actions [5]. This acknowledgement has led to a revisioning of nursing values in the 21^st^ century for example in the UK the 6C’s (care, compassion, courage, communication, commitment and competence) [8], in Ireland the 3C’s (care compassion, commitment) [9] and in Taiwan these values are stated to be humanistic caring, professionally competent holistic care, fostering growth, experiencing the give and take in caring, fair compensation, health promotion [10]. Caring for individuals with palliative care needs is an integral role of nurses. However, in more recent year’s significant changes in technology and medical advances have altered the disease trajectory and symptom management for patients, consequently changing the parameters for quality palliative care nursing [11]. This raises the question as to how nursing will maintain a balance between the art and science of nursing.

While advances in medicine, pain management, radiological and drug interventions are important and have a clear role in care provision they can also add to the complexity of care within a palliative philosophy [12]. The impact of this phenomena has significance not only for the patient but also for nurses, who are being taken away from the beside and the sensitive, caring, art of nursing [3]. The absence of the nurse at the bedside can lead to the caring aspects of, being with, being present and generally spending time with patients and families being overlooked or undervalued [7]. Given that these are important aspects of care a tension is created in current practice [3]. These developments have impacted on the role of the nurse in palliative care and leads the authors to question the role and contribution of palliative care nursing. In so doing, it is acknowledged that palliative care has continued to develop both as a concept and as a service and such growth inevitably brings changes and such changes present both opportunities and challenges for nursing. Considering the future of palliative nursing, Watts et al [13] note that nurses need to maintain their core nursing values while advancing their knowledge to encompass, clinical practice, education and research so that they can support patients and their families who are living longer with complex issues while allowing the patient to live with the best possible quality of life until they die [13,14]. The role of the nurse has long been poorly understood and understated [15]. It is imperative therefore that nurses identify clearly what it is they do and how the numerous elements of their complex role benefits patients and families. In so doing the unseen/hidden elements of nursing care are made visible and valued. Following on and building on Moran et al’s [16] work which identified the values of palliative care nursing this paper aims to describe the role and contribution of nurses providing palliative care to inform future practice in the delivery of palliative nursing, research, education, and nursing policy development.

## Methods

Due to the broad multifaceted nature of palliative care and palliative care nursing, a scoping review methodology was chosen to present a broad synthesis and mapping of the available literature centred on the review question and not limited by study quality or design [17] to identify the current body of knowledge and existing gaps in the literature [18]. Through the systematic synthesis of the evidence a rigorous map of the findings is presented [19]. To present the extent and nature of the literature, identify gaps and make recommendations Arksey and O’Malley framework [17] incorporating updates by Levac et al [20] and Bradbury-Jones et al [19] was adopted. A five-step process was utilised: i) identifying the research question, ii) identifying relevant studies, iii) study choice, iv) plotting the data, and v) arranging, summarising and communicating the outcomes. This was an interactive method where each step was revisited and advanced throughout the process [21].

### Identification of research question

The review addresses the following question: ‘what is the role and contribution of nurses in caring for patients with palliative care needs’.

In addition, this review reports identified gaps in the current evidence and makes recommendations for future research as per the PAGER framework [19]. To address the review question, the review team utilised the Oldland et al framework [22] to map the evidence onto the seven domains of professional practice: (a) medical/nursing and technical competence, (b) person centred care, (c) positive interpersonal behaviours, (d) clinical leadership and governance, (e) promotion of safety, (f) management of the environment, and (g) evidence-based practice along with the elements within each domain.

### Identification of relevant studies

To encompass the broad scope of palliative care literature, a wide range of keywords were used as search terms [17]. A search strategy and inclusion criteria guided the review (Tables 1 and 2) and the search was conducted across eight databases MEDLINE, Cumulative Index to Nursing and Allied Health Literature (CINAHL), Academic Search Complete, PsycINFO, EMBASE, Web of Science, Scopus and Cochrane Library (https://figshare.com/articles/dataset/Palliative_care_-_role_and_contribution_of_the_nurse/23968647). The search was conducted on 8th March 2023 and updated on the 30^th^ April 2024 (OD) after the search strings were developed and agreed upon by the review team. The search words were used in ‘title’ and ‘abstract’ searches utilising Boolean operators ‘OR’ and search strings were combined using Boolean operators ‘AND’. All citations were exported to Endnote Library 2021 (Clarivate Analytics, Pennsylvania, USA) for duplicates to be identified and removed (OD, MB).

**Table 1:** Database search.

	Search terms
S1	‘palliative care nurs*’ ‘palliative care clinical nurs* specialist’ ‘palliative care nurs* specialist’ ‘hospice nurs*’ ‘specialist in palliative care’
S2	‘end of life care facility’ ‘end of life care unit’ ‘hospice’ ‘specialist palliative care unit’ ‘specialist palliative care in-patient unit’
S3	‘Nurs*’ ‘care’ ‘contribution’ ‘role’ ‘function’
S4	S1 + S2+ S3

**Table 2:** Inclusion and exclusion criteria.

Inclusion Criteria	Exclusion Criteria
Nurses working in palliative care settings.	Paediatric nursing.
Nurses working in acute service areas* CCU; ED; ICU; medical/surgical units; community.	Student nurses; care assistants.
Papers where it is possible to extract data focusing on palliative care nurses or nurses working with palliative patients.	Papers referring to specific elements of the role i.e., how the nurse communicates.
Papers published in the English language.	Papers relating to specific symptoms.
Papers published prior to search date of the 30^th^ April 2024.	
Papers focusing on the role of the nurse.	

* Coronary Care Unit; Emergency Department; Intensive Care Unit.

### Study selection

**Fig 1:**
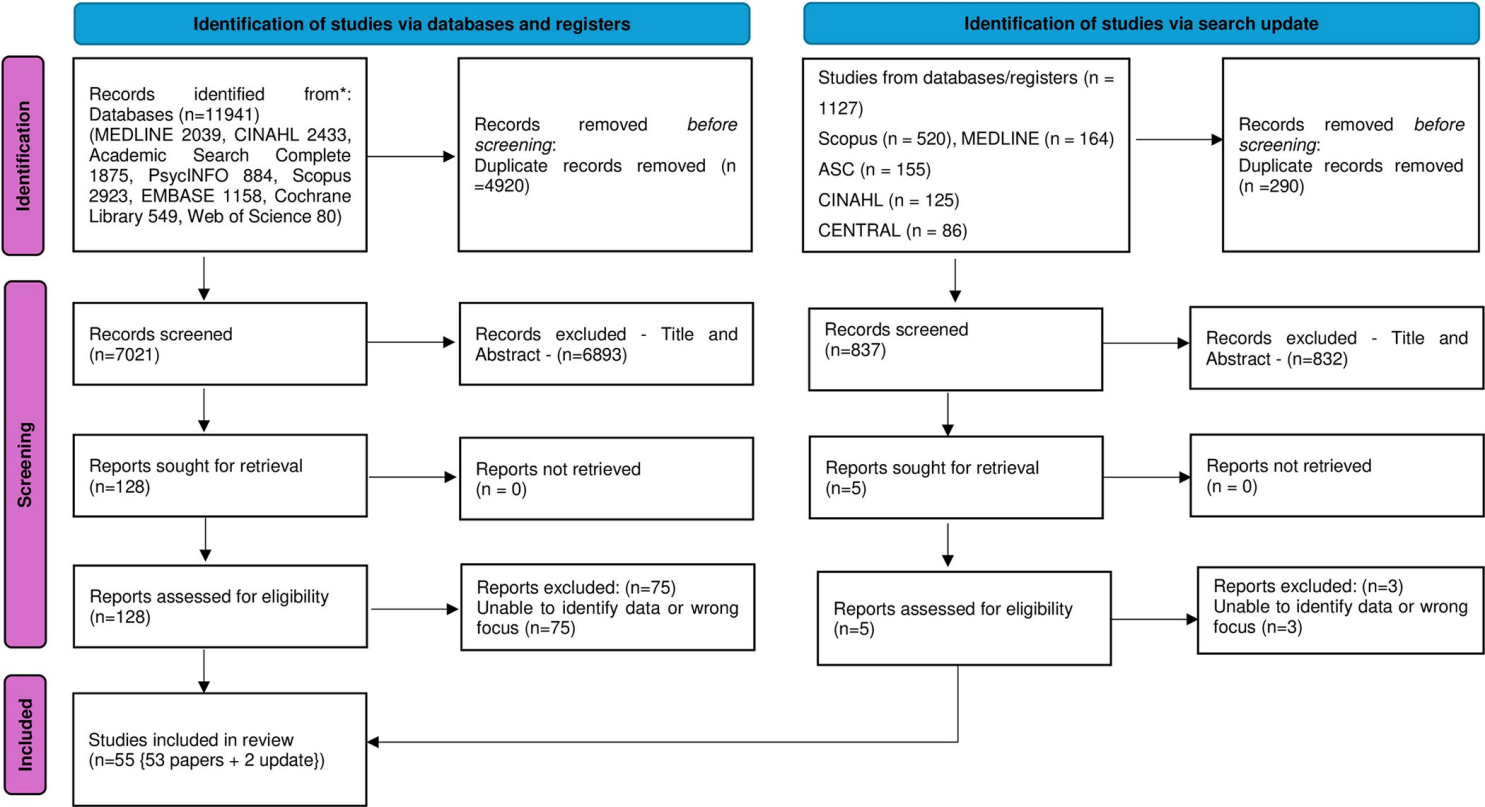
PRISMA 2020 flow diagram for searches of databases with update.

The original search generated 11,941 papers, and after the removal of duplicates (n = 4,920), 7,021 papers moved forward to title and abstract review. Screening was conducted independently within Rayyan (Qatar Computing Research Institute) by three reviewers (OD, MB, SM) against the inclusion criteria (Table 2). Following title and abstract screening, 6,893 papers were excluded, and the remaining 128 papers went forward to full-text review. The full text of the 128 papers was retrieved and screened by paired reviewers working independently (OD, MB, SM). Any disagreement or differences between reviewers were discussed with the third reviewer, and agreement was reached. Excluded reasons were recorded and reported (Fig 1) and 53 papers met the criteria for this review. The search update revealed 1127 results with 290 duplicates and 832 removed at title and abstract screening. This left 5 for full-text screening with 3 papers excluded and 2 papers meeting the criteria for this review (Fig 1).

### Mapping/Plotting of data

The data were mapped onto the framework of professional practice by Oldland et al [22]. All data pertaining to the work and contribution of palliative care nurses were extracted from each paper that met the inclusion criteria. All data extracted were coded by holding a team meeting to map all codes onto the elements and domains within Oldland et al [22] framework (S1 File). In total 776 items were coded onto 47 elements across the seven domains (S1 File). Within the coding process, it became apparent that data could span across more than one code, and to agree the coding, the review team meeting was essential to form a consensus. The coding decision process was guided by the original aim and context of the study from which the data were extracted rather than taking the highlighted data in isolation. All data extracted were synthesised and summarised using a data extraction tool agreed upon by the reviewers (S2 File).

### Arranging, summarising and communicating the outcomes

The final stage summarises and communicates the findings [17], where data were mapped onto the seven domains of Oldland et al [22] professional practice framework and conveyed through narrative in-text and tables [23]. This review is reported following the PRISMA flow diagram [24] (Fig 1) and the Preferred Reporting Items for Systematic Reviews and Meta-analysis extension for Scoping Reviews (PRISMA-ScR) checklist [25] (S3 File).

## Results

The search of the databases generated 55 papers which met the inclusion criteria. The reasons for exclusion of papers (n = 78) are reported in the PRISMA flow diagram (Fig 1). The results present the characteristics of the studies and the results of the data coding which is presented below in accordance with each domain from Oldland et al [22] framework (elements and domains). The domains are presented in numerical order to assist with the key aim of the scoping review: to map and make recommendations. Within each of the domains the individual elements from Oldland framework are also presented in terms of frequency representation of the occurrence of the element which is identified by its number of coded occurrence (n =) and to enable tracking to its source publication this is acknowledged by [reference/s]. It is acknowledged that an element may appear in a paper more than once (S1 File).

### Characteristics of the studies

The characteristics of the studies (Table 3) within this review spanned across twenty countries and were overwhelmingly qualitative, with only five out of the fifty-three studies being quantitative or mixed methods. The sample sizes of the quantitative studies ranged from 107 to 717 participants, while the mixed methods studies sample size ranged from 19 to 74, and qualitative studies sample size ranged from 1 to 129.

**Table 3:** Characteristics of the study.

**Country**	**n =**	**Paper**
United Kingdom	n = 10	26–35
Canada	n = 9	36–44
Australia	n = 6	45–50
United States of America	n = 6	51–56
China	n = 4	57–60
Sweden	n = 4	61–64
New Zealand	n = 2	65,66
South Korea	n = 2	67,68
Belgium	n = 1	69
Brazil	n = 1	70
Finland	n = 1	71
Iran	n = 1	72
Italy	n = 1	73
Japan	n = 1	74
Korea	n = 1	75
Norway	n = 1	76
South Africa	n = 1	77
Taiwan	n = 1	78
Turkey	n = 1	79
Uganda	n = 1	80
**Design**	**n =**	**Paper**
Qualitative	n = 50	26,27,29–46,48–51,53,54,56,58–80
Quantitative	n = 3	52,55,57
Mixed methods	n = 2	28,47

### Person centred care

Person centred care was described using thirteen of the fourteen elements the element not evident was equitable and accessible healthcare. For the elements evident partnership with clients and families (n = 63) [26–61] focused on the provision of support, education, training and advice to patients and families. Fundamental within the partnership was communication, knowing each other (nurse/patient/family), being there, being available, the focus on creating a bond and positive memories, and preparing the person and family. Engaging authentically (n = 63) [27–29,31,33–36,39–41,43,45,46,48,51–53,58,59,61–69] consisted of the caring relationship between the nurse and the patient and family. Key to authentic engagement was building a relationship, respecting individuality, being non-judgemental, trust, being present, listening, spending time with the person, and utilising everyday encounters to connect with the person and family. Provision of holistic care (n = 44) [30,31,34–36,40,41,43,46,49,51,53,57,58,60,62,64–67,69–72] addressed the nurses’ approach to holistic and individualised care.

This holistic approach was beyond the physical aspect of care and encompassed social, spiritual, and emotional aspects such being supportive, addressing emotional concerns, providing reassurance, offering advice and care after death, attending to the dying patients and family’s needs, dealing with practical concerns, being holistic, and supporting the family say farewell. Empathy and care (n = 24) [26,35–39,41,47,49–51,55,58,66–69,73,74] addressed being available, facilitating expression of emotions (patient and family), being empathic, supporting the person and family, being caring, listening and understanding the person’s experience, slowing down (unhurried care), making memories, protecting patient from unnecessary suffering, recognising patient being alone, and holding and being comfortable with uncertainty. Recognition of client preferences (n = 22) [26,27,29,31,37–39,45,47,51,54,67,68,72,75,76] involved meeting expectations, fulfilling the wishes of the patient, valuing the patient’s uniqueness, going at the patient’s pace, reducing distance between patient and family, helping patients to stay at home and creating an environment so patients can live as full as possible.

Ensuring spiritual well-being (n = 22) [27,31,35–37,39,41,46,48,51,53,57,61,70,74] while this is an element of holistic care, this was specifically referred to in the papers. It addressed the nurses’ awareness, sensitivity and understanding of spirituality, supporting the person to say goodbye, ensuring the patient is at peace, preserving hope and meaning, making sense of suffering, providing emotional comfort, involving community in supporting dying patients, and providing pre and post bereavement care. Promoting and respecting informed decision-making (n = 12) [26,35–37,40,46,57,58,68,69] addressed getting to know the patient wishes, involving the patient in decision making, participating in advanced care planning, respecting patient choice and individuality, negotiating choices, and facilitating breakthroughs and acceptance. Patient/client advocacy (n = 10) [31,33,40,42,45,52,56,67,73,77] focused on nurses’ advocating for patients wishes, choices, and goals of care. Maintaining dignity, privacy, and confidentiality (n = 10) [26,34,39,41,67,68] highlights nurses’ fostering respect, privacy, dignity, and autonomy, giving time and space, providing unhurried gentle care, demonstrating respect for the body and the appearance of the families loved one. Independence/empowerment (n = 9) [27,35–37,48,62,65] addressed activities that supported promoting the independence and empowerment of the person so that they could achieve greater autonomy within their care decisions.

Cultural knowledge and sensitivity (n = 8) [30,57,58,60,68,76] focused on nurses being culturally aware and sensitive, supporting religious beliefs and dealing with different cultural beliefs about hospice and dying. Open disclosure (n = 6) [31,36,48,53,68] addressed being open and honesty, being realistic, addressing misconceptions, letting the family know that death is approaching, and acknowledging death. Awareness of health literacy (n = 1) [43] focused on providing instruction regarding caregiver’s health.

### Positive interpersonal behaviours

Positive interpersonal behaviours were described using thirteen of the fourteen elements the element not evident was mutual performance monitoring. For the elements evident ownership/accountability/role responsibility (n = 53) [27,31,34–36,38,40,50,51,54,59,60,63–65,67,69,72,73,75] focused on philosophy of care and dying, valuing work and self, supporting others, preserving own integrity, accepting death, dealing with emotions, recognising limitations, caring for self, managing stress, avoiding being overwhelmed, balancing guilt and compassion, utilising time effectively, and recognising risks within the therapeutic relationship, personal and professional boundaries.

Effective communication (n = 29) [27–30,32,35–37,43,46,55,57,58,61,65,66,68,69,71,75,77–79] addressed advanced communication skills, communicating across complex levels (patient, family, professions), active listening, providing information, assessing information needs, providing honest information communicated sensitively, and having difficult conversations. Emotional intelligence and relational empathy (n = 32) [27,34–36,38,45,58,60,65,66,71,74,76,79] focused on providing a positive experience of being caring for, getting to know the family, understanding the patient’s narrative, developing a reciprocal relationship, spending time with the patient, being present, responding to anger, seeing things through the patient’s eyes and sharing secrets. Team leadership/climate teamwork orientation (n = 21) [29–32,34–36,46,50,52,58,63–65] addressed teamwork, information sharing, care sharing, clarity of team member roles, challenging treatment approaches, pushing for a decision, responding to colleagues, and discussions with the team regarding options. Adaptability (n = 16) [26,28,32,35,36,38,51,56,59,67,68,71,79] addressed the flexibility and responsiveness of the nurse and their ability to embrace challenges, having multiple tasks, roles, and responsibilities. However, there is ambiguity in roles, and in the acute setting, nurses are frequently pulled in different directions due to a lack of role clarity and crossover of roles. This results in the nurse having to explain their role and professional boundaries regarding their responsibility for caring for the gravely ill/dying patient. Collaboration (n = 16) [31,32,45,51,55,58,63,69,72,75,77–79] addressed collaborative working and liaising with others in the provision of care. This included the patient, family and healthcare team, the nurse fulfils a networking and resource role in providing information, supporting, and coordinating care. Professionalism (n = 12) [31,35,36,38,41,45,47,51,65,69] addressed the professional manner of the nurse, being prepared, their professional approach, being dedicated and committed to caring, palliative care and supporting the dying person, their passion for nursing as a caring profession, establishing credentials and personal integrity. Conflict resolution (n-11) [35–37,41,46,65,68,73] focused on resolving family and contextual conflict, diffusing and mending relationships. Reflective practice (n = 8) [35,36,40,50,69,72] addressed nurses exploring their thoughts on caring, learning from experience, examining feelings and emotions, and acknowledging one’s reactions. Mutual support (n = 5) [35,36,57] focused on creating opportunities for staff and residents in nursing homes to visit dying patient to say goodbye and valuing each person experience, feelings, and knowledge. Being ethical (n = 4) [26,35,36,39,45,55,57] addressed being honest, dealing with ethical dilemmas, being ethical, ethical competence, and doing the right thing. Situation assessment and advocacy (n = 3) [35,36,43] addressed adapting an approach according to family dynamic and focusing on living. Life-long learning (n = 1) [80] focused on ongoing learning.

### Medical/Nursing and technical competence

Medical/nursing and technical competence were described using two of the three elements the element not evident was health informatics proficiency. The first element, psychomotor skill/discipline and context-specific knowledge (n = 116) [26–28,31–39,41–47,49–53,55,57–63,66–70,72–75,77–80] addressed areas of assessment, planning/designing, implementation, and evaluation of care. Evident within psychomotor skill/discipline and context-specific knowledge were clinical, physical, social, emotional, and technical aspects of nursing care within a biopsychosocial approach.

Additionally, key aspects of observation, knowing the person, creating a plan of care based on specialised knowledge, intuition, and understanding were fundamental to supporting and preparing the person and their family on their journey. The second element, critical thinking and problem solving (n = 28) [27–29,34,37,38,43,45,49,50,54–56,59,60,67,69,71,72,75,78,79] focused on the nurses’ ability to adapt and respond to patient needs, changing needs, complex needs, decision-making, and problem solving. Nurses addressed challenges through dialogue, intuition and knowing the patient.

### Clinical leadership and governance

Clinical leadership and governance were described using all nine elements. Unit based/direct care, strategic leadership, and clinical co-ordination (n = 36) [30–33,35,36,41,45–47,55,58,59,63,69,73,75,78] portrayed the leadership and liaison role of the nurse in care co-ordination within and across services. Here nurses engage in leadership by taking charge, making arrangements, referring to others, negotiating transfers, networking, utilising resources, and putting into place e.g., medication, equipment and consultation. This demonstrates the professional and central role of the nurse operating on many levels in ensuring efficient transition of patients and discharge planning. Supervision and education of other health professionals (n = 17) [31–33,41–43,48,52,55,57,69,73] described the provision of education, teaching and training to nurses and healthcare members, supporting the development and competence of others, supporting transitioning to the work area, and providing leadership. Mentorship (n = 10) [31,42,43,49,55,60,73] highlighted the role of the nurse in guiding, supporting and mentoring fellow nurses and healthcare team members to improve care at end-of-life. There was a pedagogical underpinning here, moving between counselling and education and moving away from hands-on to consultation and support. Professional development (n = 7) [31,43,63,80] involved activities that influenced professional practice through engaging in personal and professional development, training, and education to support developing one’s experience and support professional standards. Audit/policy/guidelines/service plan (n = 2) [39,69] related to policy adherence and uphold policy and procedures. System knowledge (n = 2) [30,32] related to resource identification and management and navigating the health care system to mobilise supportive resources. Mobilising others (n = 2) [45,57] addressed building a community and mobilising action and others. Initiation, monitoring and participation in quality improvement strategies (n = 1) [75] aligning to improving practice through quality improvements. Research activity (n = 1) [73] referred to data collection for research.

### Management of the environment

Activities related to management of the environment were described using all five elements. Patient and family comfort (n = 21) [26,28,31,33,34,39,41,47,48,53,62,67,70,71] addressed providing relief, comfort, and security, avoiding suffering, adapting and adjusting the care environment, managing psychological concerns, creating a less technical atmosphere and an atmosphere of normality, and supporting the presence of the family at bedside of dying person. Patient privacy (n = 3) [47,53,62] highlighted how the nurse creates an environment that is different from the hospital, promotes an appropriate location for end-of-life, and preparing room for death.

Noise minimization (n = 2) [34,62] highlighted how the nurse works to create a calm environment and minimise noise. Appropriate lighting (n = 1) [34] highlighted how nurses adjust lighting to be more subdued to promote comfort. Clean and tidy environment (n = 1) [39] highlight how the nurse works with the physical space and the ward environment to promote comfort as best they can.

### Promotion of safety

Promotion of safety was described using three of the nine elements. The six elements not evident were error reporting, infection prevention, medication safety, safety procedures and compliance, safety culture promotion and personal safety awareness. For the elements evident understanding human and environmental factors that mitigate harm (n = 19) [27–29,39,41,43,47,49–51,59,62,73,76,79] described balancing the demands of the role and enabling a feeling of being safe and secure. Recognition and response to adverse events including clinical deterioration (n = 2) [27,33] recognised the busy workplace and the demands this may pose and supporting the patient and family following a lane change (curative to palliative care). Risk identification and management (n = 1) [28] addressed being involved early in the person palliative care journey.

### Evidence-based practice

Evidence-based practice was only described in one of the seven elements within this domain. The element evident was knowledge translation [51] which focused on the delivery of the best possible care based on evidence. The six elements absent were: formulation of clinical questions, critical appraisal and synthesis of evidence, evidence development and generation, evidence dissemination, evaluation of evidence-based decisions and practices, and understanding research and statistical terms and methods.

## Discussion

This scoping review draws together the research literature on the role and contribution of nurses in caring for patients and families with palliative care needs. This discussion addresses each domain evident within this review in the context of the wider literature.

Domain one, person centred care clearly captured the essence of palliative care highlighting the aspects of being present and sharing the journey. Relationship is key to the nursing care process (relational care) and connection is key to the relationship, with this connection developing through the therapeutic and professional use of self and the principles of empathy, trust, genuineness and congruence [81]. However, fundamental to building this relationship is the requirement of time and availability of the nurse. Evidencing these invisible core nursing functions will be critical to the continuing development of the artistic role of the nurse. Bramely and Mattti [82] report that patients expect nurses to have time for them and to listen to them however, nurses reported that a busy ward environment meant time was a precious commodity in care and compassion and when time does not exist it impacts on caring [50]. Nurses who are seen to make time for their patients are reported as being compassionate however, if a patient must wait for care due to time pressures, care is considered to be less compassionate. Several international studies in the early days of palliative care nursing; Reiman [83], Lawler [84] and Taylor [40] indicate that nurses valued relationships with patients i.e., building trust; advocacy, being there, being with.

However, within this domain the element of equitable and accessible healthcare was not evident in the literature, this may be because most papers originated from countries with established palliative care services, but it is essential that this element is taken into consideration in further research to highlight inequities, underserved populations and marginalised persons.

Domain two, positive interpersonal behaviours clearly captured the professional, collaborative and partnership working of nurses in providing palliative care that is holistic, responsive to the person, empathic and inclusive of the person and their family in care provision and decisions. However, within this domain the element of mutual performance monitoring was not evident but may be implied in the teamwork and communications elements coded. Sensitive nursing behaviours reflect a combination of attitude and nursing behaviours for example presence, listening, responding, being there, sitting at the patient’s bedside, not standing over the patient, touching the patient, timely care, holding someone’s hand, smiling at the patient, hands on clinical care i.e., washing and dressing. These action behaviours would be accompanied by compassionate behaviours including, respect, valuing, showing warmth, presence, paying attention, understanding, empathising. Kwon et al [72] described careful listening to patient and family needs; responding to patients in a manner that is suitable to their condition, quickly responding to patient problems and providing a moment to say farewell. Perry [4], while noting how nurses’ express compassion in attending to the ordinary but essential needs of their patients, recommends that the clinical environment should be designed to foster such behaviours. Nurses’ behaviours could be described as the art of what they do and how they do it. This lies within Carper’s ways of knowing [85] and results in the art and science of nursing. Often it is the minute details of how it looks in practice and how nurses describe their own role that is missing in discussion and/or recorded nursing documentation [86,87].

Domain three, medical/nursing and technical competence highlights developments in modern medicine, with new treatments effecting life expectancy. There is an increasing complexity of the illness trajectory [88] which is coupled with balancing treatment choices within a palliative approach. The medical/nursing and technical competence domain clearly demonstrates the specific nursing skills, nursing knowledge, creative thinking and problem-solving ability of nurses. Nurses in this review described this challenge as ‘changing lanes’ and noted the time required to work with the medical team to reassess, replan and communicate with patients and families. This was evident in both acute settings as patients transitioned from active treatment, aimed to prolong life, to palliative care and then within palliative care to end-of-life care. It is important that in maintaining the balance between monitoring the physical response to the disease and the psychosocial/spiritual aspects of care that time is afforded to the nurse to work with patients and families who are transitioning between care approaches. This is in keeping with Robinson et al [89] who noted that nurses are uniquely placed to support patients due to their advanced clinical skills and knowledge within a holistic nursing model of care. Part of this process is to develop and enhance nursing behaviours so nurses can “think and link” when delivering patient care through verbal and non-verbal behaviours [90].

Through this process nurses understanding, anticipating, and responding to patient care is scientifically evidenced based but also can recognise the intuitive art of nursing. This is important as the artistic nature of nursing is not always seen and therefore acknowledged and may now become visible through this process [90,91]. Critical to the identification of artistic nursing behaviours in palliative care is the recognition that they are processes within actions rather than outcomes and that these processes dramatically influence and shape care provision and patient and family care experiences. Nurses in clinical practice need to capture these processes not only in nursing documentation [16] but also within the organisational vision and ethos. The element of health informatics proficiency was not evident within this domain and this this need to be considered as this would address the skills, knowledge, and ability to evaluate the opportunities and limitations of health care technology and their impact on safety and quality of health care delivery, taking cognisance of the social, legal, ethical, and technical issues and ensuring compliance with the standards and regulations.

Domain four, clinical leadership and governance represented and highlighted the care coordination, mentoring, supervision, and leadership of the nurse in quality care provision. This domain may have scored low as these skills are not easily measured. Leadership within palliative nursing is grounded on qualities and leadership extends through all nursing roles. Leadership in palliative care is characterised by leading others with a clear vision of palliative care, motivating others to achieve quality care, positively relating to others, creating a healthy environment and working collaboratively in palliative care [92]. Nurses also possess the ability to respond to change through anticipating future care needs [93]. Care coordination can have a negative impact on family members due to the lack of clarity and ambiguity of roles resulting in family members feeling responsible for and overburdened by care coordination [94]. The relational and care coordination aspect of the nurse role helps off set this where the nurse builds a relationship with families and involves them in the care of their loved one. Care coordination brings to the fore some of the core principles of nursing, such as person centredness and advocacy [95] and ensuring the patient is in the centre rather than the system in the centre [96]. It could be argued that the art and science of palliative care nursing is particularly vulnerable to being eroded by a reductionist approach to patient care. For example, Costello [97] suggested that the art of nursing in palliative care and the time and availability of the nurse for key nursing skills such as communication based on compassion, empathy and genuine kindness are less evidenced and are being challenged by the increasing pace of busy units. This is in keeping with the findings of Moran et al [16] which highlights the challenges of time and availability to be with the patient. This emphasises that the artistic nursing skills have not only to be valued by the nurse themselves but also by nursing managers, leaders, and healthcare professionals within the care team. Nursing leadership needs to claim and value the artistic elements of nursing, support its inclusion, and showcase its outcomes.

Domain five, management of the environment may have been low in representation however all elements were evident, and consideration needs to be given to the possibility that data was only coded onto one element when in fact it may have been represented in more than one element and the decision to code was based on the context of the paper and the data originated from. The influence of the care environment is known to effect patients’ overall well-being [98] an environment that provides opportunities for social interaction and privacy are important elements of care [99].

Domain six, evidence of the promotion of safety was low with only twenty-two codes and three elements represented. This highlights the absence of the six elements of Oldland et al framework [22] specifically error reporting, infection prevention, medication safety, safety procedures and compliance, safety culture promotion and personal safety awareness. The reason for this omission may lie in the amount of time these activities take up, and while it is disappointing these activities were not evident the lack of observational studies may be a factor. The recent pandemic may draw attention to infection control and safety issues resulting in future publications, and regulation can address compliance, reporting and safety issues such as in Ireland for example national standards are being met such as restrictive practice [100] and medication administration and management [101].

Domain seven, evidence-based practice appeared to be lacking as only one of the seven elements were evident. However, this may be due to the scoping nature of the paper and the mapping of code onto a single element and domain. While it could be argued that evidence underpins practice there was no evidence of six elements within this domain which highlights an opportunity to address this gap in the literature in future studies and make these elements explicit in research pertaining to nursing in palliative care. There is a need for palliative care nursing research and for nurses to be involved in research teams. Such involvement can be in the conduct, collection, analysis, or interpretation of data. However, there is a need to develop nurses’ confidence in research, provide time within their role for research and create nursing research posts specific to palliative care nursing. The six elements absent from this domain were formulation of clinical questions, critical appraisal and synthesis of evidence, evidence development and generation, evidence dissemination, evaluation of evidence-based decisions and practices and understanding research and statistical terms and methods This highlights the importance not only for research but also in clinical practice, where there is an opportunity for nurses in palliative care to seize this opportunity and bridge this evidence gap through practice-based publications and research studies. Nurses need to be actively engaged in patient end-of-life research in both the conduct and dissemination of such research.

Davies and Oberle [36] described a model for a supportive nursing role in palliative care which consisted of valuing; connecting; empowering; doing for; finding meaning and preserving own integrity. These researchers proposed that the concept of support is complex and that the nature of support in nursing is poorly defined. This view continues to have value today. Revisions of the Davies and Oberle model [36] across the decades upholds the fundamental nursing roles with just two additional elements; influencing other professionals and displaying expertise Newton and McVicar [27]. The knowledge skills and attitude of the nurse are underpinned by the nursing values, care, compassion, and commitment. Throughout this review sensitive nursing behaviours are evident across all seven domains. Critical to the identification of artistic nursing behaviours in palliative care is the recognition that they are processes within actions rather than outcomes and that these processes dramatically influence and shape care provision and patient and family care experiences. Nurses in clinical practice need to capture these processes in nursing documentation but also within the organisational vision and ethos [16].

The findings of this review are in keeping with Sekse et al [102] and demonstrate that time is a precious and scarce commodity. The scientific workload has increased in the field of palliative care creating a ‘push me—pull you’ challenge for the nurse supporting patients with palliative care needs. The complex disease trajectory and advances in treatment has additional implications for communication skills and time. The role of the nurse is complex and difficult to clarify and, as it is often unseen work, it is frequently undervalued. Invisible care is challenging to describe and document [103] and further research in this area is required, however, from this review it is apparent that the nurse holds a key role in the support of patients with palliative care needs. Overall, Oldland’s framework [22] consisting of the seven domains and various elements can guide practice development, nursing documentation and research into the future. Palliative care nursing could adapt the framework and identify elements relevant to palliative care nursing that are essential but missing from Oldland’s framework. In this way palliative care nursing could look at developing nursing sensitive metrics rather than learning by trial and error and this could form the basis of education and a model for practice that would have transferability to caring for palliative care patients and their families in different care environments. Thereby, palliative care nurses can demonstrate their ability to verbalise their actions to inform and enhance the mastery of clinical practice [104,105]. Here there is an opportunity for nursing to lead, create and innovate within its own discipline and capture the essence of caring for the human condition.

## Recommendations

Based on the insights provided in the scoping review regarding the role and contribution of nurses in caring for patients and families with palliative care needs, several recommendations can be made for research, education, and policy development. Further research is required to explore the nuances of person-centred care in palliative nursing, particularly focusing on the impact of time availability on nurse-patient relationships and the provision of compassionate care. Explore the integration of evidence-based practice principles into palliative care nursing, addressing gaps in areas such as clinical question formulation, critical appraisal of evidence, and evidence dissemination. Educational programmes need to enhance nurses’ skills in providing person centred care, emphasising the importance of empathy, trust, and genuineness in nurse-patient relationships. Education using for example role modelling on sensitive nursing behaviours, such as active listening, timely care, and compassionate communication needs to be integrated and embedded into nursing curricula and practice. Policies should prioritise nurse-patient ratios and workload management to ensure adequate time for compassionate care delivery in palliative settings. Initiatives aimed at recognising and valuing the artistic elements of nursing, including compassionate communication and empathetic care, within organisational cultures and policies need to be supported.

## Limitations

While this review used precise and transparent methods based on study and reporting guidelines by Arksey and O’Malley [17] no quality appraisal was conducted as the focus of this review was to map the evidence. Thus, this paper only offers a descriptive account of available information. There was no patient and public involvement, and there are opportunities for engagement, potentially following published guidance on stakeholder involvement in systematic reviews [23]. Additionally, papers in this review were limited to databases which may have affected representativeness. The authors recognise that the evidence presented in the review is limited to the published literature regarding role and contribution of nurses in caring for patients with palliative care needs, and thereby the findings are limited to the scope and nature of the specific research question within each paper. Furthermore, the authors recognise that much of nursing care which is documented does not form part of research papers.

## Conclusions

This scoping review illustrates the evidence of the role and contribution of the nurse in caring for patients with palliative care needs and highlights that nurses embrace the core values of palliative care nursing while caring for the person and the family. However, in a scientific and technical era, it is essential to value and report the often unseen and underreported artistic and intuitive actions within nursing practice. This creates an opportunity for palliative care nursing to bridge this gap in future nursing documentation, research studies, and publications. For this to occur, nurse leaders need to actively promote nursing in the areas of holistic care, education, and research. Fundamental to this process is the adoption or adaptation of a framework such as Oldman’s to highlight the domains and elements to guide practice development, nursing documentation and research. By addressing the research, education, and policy recommendations, stakeholders can work towards enhancing the role of nurses in palliative care, improving patient outcomes, and ensuring compassionate and equitable care for individuals and families facing end-of-life needs.

## Supporting information

S1 File Data coding onto Oldland’s framework. (XLSX)

S2 File Data extraction table. (DOCX)

S3 File PRISMA reporting guideline for scoping review. (DOCX)
