# Supplementary material for: Role and contribution of the nurse in caring for patients with palliative care needs: A scoping review
Source: PLoS One. 2024 Aug 23;19(8):e0307188. doi: 10.1371/journal.pone.0307188 (PMC11343417; doi:10.1371/journal.pone.0307188)
Supplement: S2 File — (DOCX) [file pone.0307188.s002.docx]

**Supplementary file 1: Data Extraction Table**

| **Author; Year; Title; Country** | **Study aim** | **Design and sampling** | **Summary of main findings** |
| --- | --- | --- | --- |
| [75] Kwon and Byun (2024) Clinical experience of nurses in a consultative hospice palliative care service.  Korea | To explore the experiences of consultative hospice palliative care nurses. | Qualitative phenomenological design. 15 nurses. Data collection – interviews. Data analysis – Colaizzi’s phenomenological method. | Provide physical, psychosocial, and spiritual support, as well as pain and symptom management. Control of terminal symptoms. Empathise with family and understand and consider the perspectives of both patients and their families. Providing detailed explanations to families and ensuring they spend quality time with their loved ones. Provide counselling to patients and their families and offering support. Facilitate communication between healthcare professionals, patients, and their families. Exchange information about the patients and their families with the hospice team. collaborated on the development of care plans. Striving to improve hospice service quality. |
| [79] Sarıkahya et al (2023) Experiences and practices of nurses providing palliative and end-of-life care to oncology patients: A phenomenological study.  Turkey | To describe the experiences and practices of nurses who provide palliative and end-of-life care to oncology patients. | Qualitative phenomenological design. 12 nurses. Data collection – interviews. Data analysis – thematic content analysis. | Nurses fostered multiple learning outcomes: at the spiritual level, connection and communication with the patient and his family, and making sense of life and death. Through maintain feeling of hope, providing moral support, meeting self-care needs, pain management needs, wound care needs and providing psychological support. |
| [71] Melender et al (2022) Palliative-care nurses’ and physicians’ descriptions of the competencies needed in their working units.  Finland | To describe the most essential competencies of palliative-care nurses and physicians. | Qualitative descriptive design. 129 nurses and 64 physicians. Data collection – survey with open ended questions. Data analyses - content analysis with inductive and deductive approaches (Elo and Kyngäs, Kyngäs et al). | Nurse clinical competent, symptom management, pain management, assessment of care needs, pharmacological treatment’ and ‘implementing and evaluating clinical care. Emphasis on social interactions and providing support, as the most often needed competency in daily work. |
| [73] Angheluta et al (2020) When and how clinical nurses adjust nursing care at the end-of-life among patients with cancer: Findings from multiple focus groups.  Italy | To explore when and how nurses and healthcare assistants adjust end-of-life care to ensure patient’s comfort at the end of their lives. | Qualitative descriptive design. N=41 consisting of 25 nurses and 16 nurse assistants. Data collection – focus groups. Data analyses - thematic analysis Braun and Clarke. | The adjusting of the nursing care plan according to the lived experience of nursing is based on the “when” and “how.” The “when” was based on detecting the turning point and being ready to change continuously until end-of-life. The “how” was based on weighing the harms and benefits of interventions, advocating patient wishes, sharing the adjustments within the team, involving the family and allowing care to move away from evidence-based interventional care and accepting end-of-life. Early detection of the terminal phase results in care changes and improved patient outcomes. |
| [57] Chan et al (2020) Nurses perceptions of and barriers to the optimal end of life care in hospitals: a cross sectional study  China | To assess nurses’ perceptions of what constitutes optimal end-of-life care in hospital and evaluate perceived barriers to end-of-life care delivery | Quantitative survey design. 175 nurses. Data collection – survey. Data analysis – statistical analysis SPSS. | Getting to know patient and facilitates patient’s wishes. Patients’ emotional concerns are identified and managed. Advocating for patient and support participation in decision making. Attending to dying patient/family needs. End-of-life care documents. Provision of single rooms private rooms. Barriers: doctors too busy; nurses too busy; families with unrealistic expectations of prognosis. |
| [67] Kwon et al (2020) Nurses’ experiences of providing “sensitive nursing care” for terminally ill individuals with cancer: A qualitative study.  South Korea | To explore the sensitive nursing care provided by nurses who care for terminally ill individuals with cancer. | Qualitative design Colaizzi's ‘descriptive phenomenology. 20 nurses. Data collection - 37 interviews. Data analyses - Colaizzi's framework. | Teamwork and role modelling which assists others caring for terminally ill patients to improve sensitive nursing care. Nurses display sensitive attitudes and behaviour, including reflecting on past experiences, developing an accepting attitude toward death, using intuition to address critical situations, having an open mind regarding collaborating with colleagues, listening to the patient’s needs, responding to patients in a manner suitable to their conditions, quickly responding to patient problems, and providing a moment to say farewell. |
| [61] Hemberg and Bergdahl (2019) Cocreation as a caring phenomenon: Nurses’ experiences in palliative home care.  Sweden | To explore how cocreation can be experienced as a phenomenon by nurses working in palliative home care. | Qualitative hermeneutical Gadamer design. 12 nurses. Data collection – interviews. Data analyses – thematic analysis guided by Braun and Clarke. | Cocreation is seen as a fundamental part of caring, involves the profound endeavour of being deeply involved in humans’ health and holistic care. Seen as the cornerstone for realising a meaningful life for patients/families. Creates a closeness for providing a sense of security and comfort, involves teaching and encouraging aspects in a relationship. Preserves hope and meaning by being realistic and fulfils and realises needs and wishes at end of life. |
| [74] Nasu et al (2019) Rebuilding and guiding a care community: A grounded theory of end-of-life nursing care practice in long-term care settings.  Japan | To investigate the end-of-life nursing care practices process in long-term settings for older adults in Japan | Qualitative grounded-theory design Corbin and Strauss. 22 nurses. Data collection – interviews. Data analyses – guided by Corbin and Strauss. | Support dying as a social human being surrounded by people. Responding to physical and psychological distress. Nursing expertise, healthcare skills and leadership qualities. Providing care, assessing patients gaining an understanding of the diseases and the person’s life history and detecting minor changes. Harmonising care with the dying process and adjusting care strategies. Upholding patient wishes and supporting patients/families. Helping community members care, support eating or drinking, bathing, and encouraging to discuss their memories. |
| [51] Ferguson (2018) Ways of knowing and caring used by nurses in community hospice agencies.  United States of America | To explore nurses’ experiences of caring for individuals who are receiving hospice care. | Qualitative descriptive phenomenology design. 14 nurses. Data collection – interviews. Data analyses - Giorgi’s process. | Need to make difficult to-measure concepts such as knowing and caring visible so end-of-life nursing care is valued, identified, and validated. Knowing and caring add value and demonstrate a contribution to patient care. Caring is meaningful to patients/families. Provides holistic care, have a special relationship with patient/family. Nurses use different types of knowing; knowing about diseases, knowing from experience, and knowing from patient/family as persons and add to the relationships with patient/family. |
| [52] Cagle et al (2017) Caring for dying patients in the nursing home: Voices from frontline nursing home staff.  United States of America | To describe positive and negative experiences related to caring for dying patients. | Quantitative survey design. 707 participants of which 107 nurses, 282 certified nursing assistants, 211 licensed practical nurses, 27 social workers, 63 other and 17 unidentified. Data collection – survey open ended questions. Data analysis – statistical analysis and constant-comparative analysis of open questions. | Need for improving staff knowledge, coordination of care and social supports for patients. Patients experience good care and being involved in care, being prepared, family being supported, good symptom management. Staff felt honoured taking care of patients and getting to know patient/family. Negative patient experience being alone, privacy ignored, suboptimal care, family patient suffering, poor communication, resistance to end-of-life care, staff feeling helpless, not being present for patient, distressing symptoms, family conflict and overlapping roles with hospice. |
| [70] Salum et al (2017) The process of death and dying: challenges in nursing care for patients and family members.  Brazil | To understand the actions and interactions performed by nurses in caring for patients and family members. | Qualitative grounded theory design. 18 participants, 9 nurses, 6 undergraduate nursing students, 3 nursing professors. Data collection – interviews. Data analyses – constant comparative analysis process | Nurses are faced with the complexity of care in the process of death and dying. Care extends to care of the family members; understanding the uniqueness and individuality of each human being; seeking strategies for coping with death and dying. Factors supporting care is education regarding the death-dying process, the nurse-patient bond, family members support, and respect for the grieving process. Empathy is a key factor but often a challenge, considering the influence of personal and bureaucratic factors. |
| [76] Svendsen et al (2017) Dying patients in nursing homes: nurses provide “more of everything “and are “left to deal with everything on their own”.  Norway | To describe the nurses’ experience of dying patients in nursing homes. | Qualitative exploratory descriptive design. 12 nurses. Data collection – focus group interviews. Data analyses – content analysis guided by Gadamer’s hermeneutics. | Nurses care focused on being present, observations and actions. Monito patients’ pain, fear, and symptoms. Provide holistic care and treatment. It can be emotionally challenging to look after dying patients and their families, providing clarifications and planning with families. Nurses must contend with their own emotions and conscience. Nurses are professionally competent to make assessments and convey information. Nurses often pulled in different directions, have multiple tasks, roles and responsibilities when caring for the patient/family. |
| [62] Andersson et al (2016) To be involved: A qualitative study of nurses' experiences of caring for dying patients.  Sweden | To describe nurses' experiences of caring for dying patients in surgical wards. | Qualitative descriptive design. 6 nurses. Data collection – interviews.  Data analysis - content analysis Graneheim and Lundman. | Supervision is a valuable tool for bridging the gap between theory and practice in nursing. Improved knowledge about palliative care and committed nursing leadership facilitated preparation for end-of-life situations. Nurses are often personally affected with evidence of caring encompassing being supportive, being frustrated, and being sensitive in the caring process. Nurses ill prepared to care for dying patients due to lack of palliative care knowledge and their experiences could be described as a process of transition from theory to practice by trial and error. |
| [72] Ghaljeh et al (2016) Compassion and care at the end of life: Oncology nurses' experiences in South-East Iran.  Iran | To explore nurses’ experiences of caring for dying patients. | Qualitative phenomenological hermeneutic design. 10 nurses. Data collection – interviews. Data analyses - phenomenological hermeneutics guided by Ricoeur. | Nurse sees the patient as a unique person understanding that culture, belief and religion are important dimensions of human personality. Being present and seeing patients as unique individual’s enable nurses to develop a close relationship with the dying patient and support meeting their needs and preferences to provide a dignified death. Personal and professional development caused by closeness to the patent/family, can have both positive and negative experiences for nurses on how they manage these issues to maintain their own integrity. |
| [36] Oliveira et al (2016) Battling a tangled web: the lived experience of nurses providing end-of-life care on an acute medical unit.  Canada | To understand the lived experience of nurses on a medical unit providing end-of-life care to patients | Qualitative hermeneutic phenomenological design. 10 nurses. Data collection – interviews. Data analyses – qualitative analysis guided by Van Manen. | Nurses are continually battling a tangled web which represented their struggles in attempting to provide end-of-life care in an environment that is not always conducive. Caring is complex and nurses bear witness to suffering, striving to create comfort for the patient and work through the dying process with the family. Often nurses are left struggling through the process. |
| [37] Reimer-Kirkham et al (2016) ‘Close to’ a palliative approach: nurses’ and care aides’ descriptions of caring for people with advancing chronic life-limiting conditions.  Canada | To explore nurses’ and nursing assistants’ perspectives of a palliative approach in a variety of nursing care settings. | Qualitative interpretive descriptive design. 25 nurses and 5 nursing assistants. Data collection - interviews and focus groups. Data analyses - thematic analysis. | Nurses committed to providing better end-of-life care, and they understood palliative approach as an extension of specialised palliative care services. Participants varied in their self-reported capacity to integrate a palliative approach, as they were influenced by role clarity, interprofessional collaboration and knowledge. |
| [58] Tse (2016) Emergency nurses perceptions of providing end of life care in a Hong Kong emergency department: a qualitative study.  China | To explore emergency nurses’ perceptions regarding the provision of end-of-life care in the emergency department. | Qualitative approach not specified. 16 nurses. Data collection – interviews. Data analyses - content analysis | Nurses focus on doing good for dying patients, estimate end-of-life care, provide a desirable location for end-of-life, provide comfort and care for end-of-life patients. Facilitate family engagement and involvement in choosing intervention options; supporting the family in expressing their concerns and love to the end-of-life patient; balancing family needs and patients’ interactions and dignity while dying and during the farewell. Care is enhanced by personal growth and professionalism and challenged by resources, manpower, service priority and resource utilisation. |
| [77] Brysiewicz and Campbell (2015) The uniqueness of care: Nurses’ stories of providing palliative care.  South Africa | To explore the experiences of nurses in providing palliative care to patients dying of a terminal illness in a hospice. | Qualitative narrative design. 3 nurses. Data collection – interviews. Data analyses - thematic analysis Reissman. | Pain management is central. Challenges as to when to use morphine, difficulty in relieving emotional pain, and negotiating with the doctor regarding dosage. Doctors and nurses need to work together. Celebrating living is important and creating opportunities to enjoy time with loved ones, to still enjoy life and make memories. Caring in different ways “treat this place as home” enabling loved ones stay overnight and engaging spiritual care to see what gives them strength. |
| [53] Arbour and Wiegand (2014) Self-described nursing roles experienced during care of dying patients and their families. A phenomenological study.  United States of America | To improve understanding of the role perceptions experienced by critical care nursing during transition from aggressive, life-saving care to palliative and end of life care. | Qualitative descriptive, phenomenological design. 19 nurses (critical care). Data collection – interviews. Data analyses - Coliazzi’s method of data analysis. | Nurses support and educate the family including on withdrawal of treatment. Advocate for the patient through documenting patient wishes. Encourage and support family presence and allow the family to say goodbye. Managing symptoms such as pain control and supporting comfort. Promoting family centred care by supporting, protecting and creating positive memories. Mentoring and teaching of novice staff. There is a need for future research exploring best practices to mentor, teach, and prepare nurses to provide best practice at end-of-life care. |
| [26] Efstathiou and Walker (2014) Intensive care nurses’ experiences of providing end-of-life care after treatment withdrawal: a qualitative study.  United Kingdom | To explore the experiences of intensive care nurses provided end-of-life after a decision had been taken to withdraw treatment. | Qualitative descriptive design. 13 nurses (intensive care). Data collection – interviews. Data analyses - analysed using the principles of interpretative phenomenological analysis. | Nurses’ ‘doing the best to facilitate a comfortable and dignified death’ focusing on the dying patient/family; providing and encouraging presence; reconnecting the patient and family; and dealing with emotions and ambiguity. Uncertainties exists on how to reduce the technological environment. Caring involved physical care and symptom management, providing, and encouraging presence. Reconnecting the patient and family involved creating a less technical environment; reducing technical care; reducing distance between patient and family; increasing privacy and proximity. Dealing with emotions, ambiguity, uncertainties, or relations based on what is the right thing to do. |
| [27] Howell et al (2014) Community palliative care clinical nurse specialists: a descriptive study of nurse – patient interactions.  United Kingdom | To describe community palliative care clinical nurse specialist’s activities during interactions with patients. | Qualitative design not specified. 4 nurse specialists. Data collection – observations and audio-recording of interactions, observed during 38 interactions with 34 patients. Data analyses - thematic analysis. | Engaged in nursing assessment, planning, intervention, and evaluation. Operate core principles of palliative care, autonomy, communication, and a multi-professional approach to care. Deal with complex multifaceted problems, real time decision making, leadership and coordination of care. Respond to complex and varied situations and communication techniques. Provide comfort and care. Patient should have a dignified death and not die alone. Conflict a source of frustration with moral and ethical dilemmas. Nurses struggled with medical decisions that appeared to exacerbate or exposed patients to unnecessary suffering. Delivery of emotional and physical peace and deal with environment and organisational issues. |
| [68] Kim et al (2014) Nursing home nurses’ ways of knowing about peaceful deaths in end-of-life care of residents: Personal knowledge and strategies.  South Korea | To explore nursing home nurses’ ways of knowing about peaceful deaths at end-of-life care for residents. | Qualitative exploratory design. 21 nurses. Data collection – interviews. Data analyses – conventional content analysis unspecified. | Nurses aid patients achieve a peaceful death. Recognise subtle changes in baseline such as changes in muscle tone, abilities, excretion, and blood circulation. Value the importance of nesting which entails preparing the place for death, providing familiar private room. Comfort relief from distressing symptoms, providing emotional and spiritual support. Promote fulfilment by meeting missing people, spending the final moment with family and maintain presence through preserving self-esteem through the partnership until the end. |
| [28] Newton and McVickar (2014) Evaluation of the currency of the Davies and Oberle (1990) model of supportive care in specialist and specialised palliative care settings in England  United Kingdom | To evaluate the extent to which the Davies and Oberle (1990) model of supportive nursing care has currency in specialist and specialised care settings. | Mixed methods design in four stages. Phase 1 25 nurses, phase 2 48 nurses, phase 3 0 nurses, phase 4 19 nurses. Data collection – focus groups, survey, interviews. Data analyses –qualitative analysis unspecified and survey statistical analysis and open-ended question analysed utilising Colaizzi’s framework. | Connection with the patient is affected by pace of work and lateness of referrals. Spending time is increasingly difficult to sustain and service delivery continues to change. Nurses connect with the patients/cares occurs through getting to know each other in a deep sense. Provide physical care, and support the patient find meaning through strength giving, helping the patient to live as fully as possible until death but also preparing for death. Nurse display expertise and influence other professionals through advanced communication skills and prioritising agreeing the plan. Nurses empower patients by retaining independence for as long as possible and in valuing each patient. Nurse supports the intrinsic worth of humanity in particular aspects of each person. However, preserving their own integrity is an issue from a professional and a person perspective. |
| [65] Raphael et al (2014) The role of practice nurses in providing palliative and end-of-life care to older patients with long-term conditions.  New Zealand | To explore the role of practice nurses in the provision of palliative and end-of-life care to older patients with long-term conditions. | Qualitative, descriptive design. 21 nurses. Data collection - interviews. Data analyses - thematic analysis Braun and Clarke. | Practice nurses limited education and training in end-of-life care and involvement influenced by the context in which they work, their role and whether the GP took the lead and involved them in the patients’ care and management. Barriers include a lack of time and opportunity to attend training and not being funded for training. Nurses spend with patients are a point of contact and can coordinator other services. Nurses provide supportive care, build a relationship with patients to ascertain their needs, advocate for patients and deliver a patient-centred approach. Coordination ensured continuity of care. |
| [54] Ellington et al (2013) Complexities for hospice nurses in supporting family caregivers: Opinions from U.S. thought leaders.  United States of America | To explore the challenges in supporting family caregivers in home hospice | Qualitative design unspecified. 11 participants unspecified (nurse researchers, clinical leaders, hospice / palliative nurse educators, national leaders in hospice, symptom management / palliative care). Data collection – interviews. Data analyses – qualitative analysis unspecified. | Tensions and complexities exist for nurse supporting home hospice family and caregivers. Caregivers needs often change overtime and vary for different family members increasing complexity of nurse assessment as patient’s status changes. Family centered homecare requires nurse to teach and support the family in providing most of the hands-on care which requires an understanding of complex family dynamics. Pain or anxiety management is essential to support the family caring. Skilled communication by the nurse is required to adapt to the wide variety of situations and engage in skilled listening to synthesise multiple sources and types of information. Support a continual transition where nurses use their knowledge and expertise to act as navigators for family caregivers to understand what to expect as the patient’s condition changes. |
| [59] Mak et al (2013) Experiences and perceptions of nurses caring for dying patients and families in the acute medical admission setting.  China | To explore nurses caring for dying patients and their families in the acute medical admission setting. | Qualitative interpretive descriptive design. 15 nurses. Data collection – interviews. Data analyses - thematic analysis Boyatzis’s. | Caring for dying patients can exert strain on the nurses. Lack of preparedness for sudden unexpected death; coping with family’s responses to patients’ deaths; nurses blamed and challenged by families. Nurses experience self-blame, disappointment and feelings of inadequacy, and disappointed at being task orientated and adhering to inflexible hospital rules. Many satisfied with their roles despite sadness at the outcome. To cope with own emotions some nurses described avoided relatives of dying patients in attempt to disconnect. |
| [45] McCallum and McConigley (2013) Nurses’ perceptions of caring for dying patients in an open critical care unit: a descriptive exploratory study.  Australia | To describe the provision of end-of-life care in an open high-dependency unit. | Qualitative, descriptive, exploratory design. 5 nurses. Data collection – interviews. Data analyses – thematic analysis Braun and Clarke. | Provide comfort and care, nurture and protect from unnecessary suffering. Adequate pain relief and symptom management. Protecting the patients’ dignity, providing physical care and basic nursing duties (pressure, eye, mouth care). Ensuring the person does not die alone and has a dignified death. Caring for the family and supporting other patients. Moral and ethical dilemmas associated with medical management, particularly when it will not change a patient’s outcome. Providing emotional and physical peace and advocating guided by patient beliefs and adapting the environment. |
| [29] Balasubramanian and Read (2012) Hospice nurse’s perceptions of caring for patients with a non-malignant diagnosis: A single case study.  United Kingdom | To explore nurses’ perceptions of caring for patients with non-malignant disease in a hospice setting. | Qualitative design unspecified. 16 nurses. Data collection - focus group interviews. Data analyses – content analysis unspecified. | Nurse focuses on experiences, collaborative working, patient perspectives, professional perspectives, knowledge and challenges. There is a need to consider the importance of timely educational preparation, proactive thinking regarding shifting medical profiles of health care, and the need for hospice managers to critically consider existing infrastructures (including supervision and support) in anticipation of diverse patient populations. Collaboration remains key to effective support. |
| [66] McCallin (2011) Moderated guiding: a grounded theory of nursing practice in end-of-life care.  New Zealand | To generate a grounded theory of nursing practice in end-of-life care. | Qualitative design Glaser’s method of grounded theory. 30 nurses. Data collection – interviews. Data analyses - constant comparative analysis process. | Nurses used moderated guiding to manage different expectations which included checking out, assessment of who knows what?. Identifying information gaps, involving and supporting patient advocacy, questioning, family inclusion, negotiating choices. Supporting professional judgement, professional boundaries, family dynamics. Safeguarding, connecting, holding uncertainty, facilitating, cultural sensitivity, backing off and creating space, guiding. Moderation required as tensions exist in patient control issues, resource constraints and nurse’s emotional investment in the nurse-patient relationship. |
| [80] Schaepe et al (2011) A spider in the web: role of the palliative care nurse specialist in Uganda: An ethnographic field study.  Uganda | To explore the role of the palliative care nurse specialist in Uganda. | Ethnographic field study design. 20 nurse specialists. Data collection – observations during home care visits, 19 days were observed and individual and small group interviews. Data analyses - thematic content analysis Burnard. | Nurses deliver holistic care to patients and their families. Holistic care involves, physical care, psychosocial care, spiritual care, providing information, networking, advocacy, and networking with family and with other professionals. |
| [63] Johansson and Lindahl (2010) Moving between rooms – moving between life and death: nurses’ experiences of caring for terminally ill patients in hospitals.  Sweden | To explore the meanings of generalist registered nurses’ experiences of caring for palliative care patients on general wards in hospitals | Qualitative, descriptive and interpretive design. 8 nurses. Data collection – interviews. Data analyses - phenomenological hermeneutical approach inspired by Ricoeur’s philosophy. | Nurses valued the role and feel complete. Self-care was an issue, having to strike a balance as empathetic caring costs energy. Nurse availability and closeness is a prerequisite to getting to know the patient/family. Time that does not exist impacts on caring. Providing palliative care in hospital and acute setting was interpreted as being about contrasts, contradictions and nurses varying attitudes as they moved between patients’ rooms. Nurses were strongly committed to be part of being part of the patient’s life journey in the best possible way despite it being stressful. |
| [55] Reinke et al (2010) Nurses’ identification of important yet under-utilized end-of-life care skills for patients with life-limiting or terminal illnesses.  United States of America | To identify nurses’ perspectives on nursing skills that are important yet under-utilized in end-of-life care | Quantitative, survey design. 717 nurses. Data collection – used survey instrument (physician skills at providing end-of-life care - Engelberg et al. Data analyses - statistical analysis and content analysis of open-ended questions. | Nurses end-of life care skills included communication skills, symptom management, competencies especially those concerning anxiety and depression, and issues related to patient-centered care systems. Nurses view their skills as important for quality end-of life care. Unmet end-of-life care educational needs and health care systems deficits prevent delivery of optimal end-of life care. There is a need for clarity of team members’ roles and interdisciplinary communication as this is important to quality end-of-life care. |
| [38] Arnaert and Wainwright (2009) Providing care and sharing expertise: Reflections of nurse-specialists in palliative home care.  Canada | To explore nurse-specialists in palliative home care. | Qualitative explorative design. 5 nurse specialists. Data collection – interviews. Data analyses - constant comparative method | Nurse specialists play a key role in interacting with family members, working collaboratively, sharing information, guiding home care nurses, being non-judgemental and building a collaborative partnership. There is a need for nurses to maintain self-care through acknowledging their own limitations. Nurses have a shared ideal and focus on making a difference. The knowledge and expertise of the nurse is related to quality care for the patient/family. Nurse specialists play a crucial role in influencing nursing practice in palliative care. |
| [46] O'Connor and Peters (2009) Palliative care nurse consultants in acute hospitals in Australia.  Australia | To explore the effect of the palliative care nurse consultant role on hospital systems and care. | Qualitative design unspecified. 11 managers. Data collection – interviews. Data analyses – qualitative analysis guided by van Manen. | There is a need for the palliative care nurse consultant in acute setting and to gather information on their practice to serve as evidence to ensure appropriate direction in future planning. The palliative care nurse consultant is a highly experienced clinician performing a key role as they smooth the way to appropriate services for patients requiring palliative care. They are senior nurses, valued for their expertise and for providing advice and education to staff. |
| [56] Pavlish and Ceronsky (2009) Oncology nurses' perceptions of nursing roles and professional attributes in palliative care.  United States of America | To explore oncology nurses’ perspectives of palliative care through narrative analysis of participants’ descriptions of life experiences. | Qualitative design not specified. 33 nurses (oncology). Data collection - focus groups. Data analyses - categorical-content narrative analysis. | Nursing roles, teaching, caring, coordinating, advocating and mobilising. Professional attributes, clinical expertise, honesty, family orientation, perceptive attentiveness, presence, collaboration and deliberateness. Nurses assist families to relate to a dying loved one and build relationships between patient and family. Perceptive attentiveness where nurses tune into patients and family, listening and investigating who the patient is by just sitting, listening and being present. |
| [78] Wu and Volker (2009) Living with death and dying: The experience of Taiwanese hospice nurses.  Taiwan | To explore the experiences of Taiwanese nurses who care for patients who die in hospice settings | Qualitative hermeneutic, phenomenological design. 14 nurses. Data collection – interviews. Data analyses – Colaizzi’s framework. | Through a good rapport with patient’s nurses assist patients with their search for meaning in life, enrich their humanity in the face of death and enrich their life experiences. The value of a trusting nurse-patient relationship is essential. Nurses provide holistic, meaningful care through close relationship with the patient and their families. Nurses often must confront and manage negative beliefs about hospice (people’s opinions and cultural). Nurse manages the dying process by fulfilling the wishes of actively dying patients and their families but must live with challenges and manage their stress and workload, and the emotional impact of the job. However, there is positive feedback from dying patients and their families, which creates feelings of happiness and energy creating a sense of self-worth. |
| [47] Canning et al (2007) Therapeutic relationships in specialist palliative care nursing practice.  Australia | To develop a competency framework to describe the core domains of specialist palliative care nursing. | Mixed method (literature review, survey including practice exemplars, interviews). 74 nurses (specialist palliative care unit) surveyed and 9 interviewed. Data collection - survey and interviews. Data analyses – statistical analysis and thematic analysis. | Therapeutic relationships central to palliative care nursing and a critical foundation for nursing practice. Shown through timing, sensitivity building trust, demonstrating respect, facilitation and maintenance of a trusting and person-centred relationship. Nurses are perceptive, sensitive and focused on patient’s needs. Nurses respond to and advocate on behalf of patients and have a deep knowledge of a patients experience or response to a life-limiting disease and impending death. Information sharing between nurses, patients and families with respect for patients’ preferences. Nurses aware of social, emotional, spiritual, and cultural factors influencing therapeutic relationships. Nurses identify patient’s goals, priorities, choices, and advocating on their behalf. Nurses use tacit knowledge and various means of evidence to guide their practice. Nurses use a variety of strategies to optimise selfcare but there is an absence of clinical supervision. |
| [30] Evans and Hallett (2007) Living with dying: a hermeneutic phenomenological study of the work of hospice nurses.  United Kingdom | To explore the meaning of comfort care. | Qualitative hermeneutic phenomenological design. 15 nurses. Data collection - interviews. Data analyses – guided by phenomenological and hermeneutic Colaizzi and Van Manen. | Nurse provides comfort care and have many complex realities within their work. Nurses ensure comfort and relief by managing the patients’ complex symptoms. Place the person at peace and ease by helping them to have a feeling of calm and relaxation, a sense of rest and freedom from the plethora of feeling and emotions that bombard patients. Support spirituality and meaning by enabling patients to find spiritual comfort and address emotional anguish. |
| [31] Griffiths et al (2007) Supporting cancer patients with palliative care needs: District nurses' role perceptions.  United Kingdom | To explore district nurses’ perceptions of their role in supporting palliative care cancer patients. | Qualitative design unspecified. 34 nurses. Data collection – interviews. Data analyses – qualitative analysis unspecified. | Nurses provide supportive and palliative care. Education alone is unlikely to improve practice without an understanding of the tensions faced by nurses in their work. Ambiguity exists in nurse’s supportive role in early palliative care. Nurses need to make contact early to support cancer patients/family. Lack of confidence and perceived skill deficits presented dilemmas that are difficult to resolve. |
| [64] Wallerstedt and Andershed (2007) Caring for dying patients outside special palliative care settings: Experiences from a nursing perspective.  Sweden. | To describe nurses’ experiences in caring for gravely ill and dying patients outside special palliative care settings. | Qualitative phenomenological approach. 9 nurses (primary home care, community care, hospital care). Data collection – interviews. Data analyses – qualitative analysis Giorgi. | The role and function of the nurse can be multifaceted and complex. Nurses pressed for time in combining care of patients in a palliative phase and those in a curative phase. On the other hand, care of healthier patients can probably give nurses positive feelings, energy, and hope in a mixed work situation. Nurses frequently have to switch between dying patients and almost-cured patients. |
| [48] Barnard et al (2006) Going on a journey: understanding palliative care nursing.  Australia | To describe how nurses understood their experience of being a palliative care nurse. | Qualitative phenomenological. 10 nurses (specialist palliative care unit). Data collection -interviews. Data analyses – analysis guided by Dahlgren and Fallsberg framework. | Nurses focus on what they can do, develop closeness, work as a team, create meaning about life, and maintaining self. Nurses strive to meet care requirements of each patient, providing hygiene care, comfort, symptom management, pain relief, emotional support patient/family, and maintain independence and control. Honest in answering questions and focus on maintaining physical comfort and dignity through open communication and symptom control. Nurses assume a nurturing role. Closeness is developed through the nurse/patient relationship. Working as a team enables nurses to provide care and support to patients/families. Nurses create meaning about life through understanding the patients experience and accepting death as a spiritual form of life. Nurses own personal values and beliefs assist them in working in palliative care.Nursing tasks and meaningful relationships are central and the deep relationships between nurses, patients, and families and the emotional nature of palliative care places a burden and demand on nurses. |
| [32] Chapple et al (2006) The specialist palliative care nurse: A qualitative study of the patients’ perspective.  United Kingdom | To explore perceptions of a wide range of issues that concern people facing death, for a website | Qualitative interpretive design guided by grounded theory and constant comparison analysis. 41 patients. Data collection – interviews. Data analyses - thematic analysis with constant comparison. | Nurses highly valued by those who have a terminal illness. Involved from time of diagnosis. Patients valued the nurses work, particularly their advice on practical matters, information provided about their disease, emotional support, advice on symptoms, and help with communication. Some patients did not realise that specialist palliative nurses may be involved at a relatively early stage in a person’s illness, and sometimes felt distressed by an early referral. |
| [33] Johnston and Smith (2006) Nurses’ and patients’ perceptions of expert palliative nursing care.  United Kingdom | To explore the perceptions of patients and nurses of palliative care. | Qualitative phenomenological design. 22 nurses and 22 patients. Data collection -interviews. Data analyses - Colaizzi’s framework in conjunction with Miles and Huberman’s content analysis process. | Palliative care nurses experienced both effective and ineffective interpersonal communication, build therapeutic relationships and support control of patients’ pain and distressing symptoms. Interpersonal skills and qualities such as kindness, warmth, compassion, and genuineness. Great value is placed in interpersonal skills and the value of the nurse-patient relationship to support patients maintaining their independence and fighting spirit. Nurses meet needs by knowing about illness, providing comfort, being there and supporting, the importance of the atmosphere, safety and sanctuary of the environment for patients to feel safe and secure. |
| [39] Thompson et al (2006) Nurses’ perceptions of quality end-of-life care on an acute medical ward.  Canada | To explore nurses’ perspective of working in an acute care setting and generate a conceptual model of nursing behaviours and social processes. | Qualitative grounded theory design. 10 nurses. Data collection -interviews. Data analyses - constant comparative method Hutchinson and Wilson. | Nurses provide high quality end-of-life care but are being pulled in many directions. Nurses create a haven for safe passage representing a continuum of behaviours and strategies and includes facilitating and maintain a lane change; getting what’s needed; being there; and manipulating the care environment. Providing quality end-of-life care is a complex involving many factors related to the patient, family, healthcare providers and the care environment. |
| [34] Kennedy (2005) District nursing support for patients with cancer requiring palliative care.  United Kingdom | To explore the role of district nurses caring for patients with cancer who require palliative care. | Qualitative case study design. 3 nurses. Data collection - observation (n=11) and in-depth interviews (n=12). Data analyses – thematic and further analysed using an interpretive strategy Hammersley and Atkinson. | Nurses are a lynch pin or coordinator in palliative care and the main providers of physical and emotional support for patients and families. Patient and carer preferences impacted on decision making and care planning. Compromise is often required. Time required to get to know the patient/family to establish relationships and establish who knows what and getting the balance right. Role involved referral to other agencies. Role also involved referral to other agencies. |
| [35] Hamilton and McDowell (2004) Identifying the palliative care role of the nurse working in community hospitals: an exploratory study.  United Kingdom | To explore nurse’s role in providing palliative care in community hospital. | Qualitative exploratory/descriptive design. 4 nurses and 2 GPs. Data collection – interviews. Data analyses - thematic analysis Morse and Field. | Nurses are involved in, communication, teamwork and relationships, holistic care, resource provision, cultural care, professional role development. The nurse’s role is multifaceted and professional, organisational and personal barriers are potential constraints. Nurse place significance on being there with the patient. Key aspects of the nurse role are caring, liaison, coordination of patient care, facilitation of communication, being the link between patient’s, carers and the healthcare team and facilitation of symptom control. |
| [60] Mok et al (2004) Nurse patient relationships in palliative care.  China | To explore nurse–patient relationships in the context of palliative care. | Hermeneutic phenomenology design Van Manen’s. 10 nurses and 10 patients. Data collection -interviews. Data analyses - thematic analysis hermeneutic process. | Reducing suffering through the provision of maximum comfort, meeting patient and family expectations and the provision of time and space. Care given is beyond superficial. Responding to patient needs quickly and in a timely and appropriate manner. Showing patients and families that they care. Developing a trusted and connected relationship. Understanding patient needs, displaying caring attitude and action, providing holistic care, acting as patient advocate. |
| [69] Georges et al (2002) Being a palliative care nurse in an academic hospital: a qualitative study about nurses’ perceptions of palliative care nursing.  Belgium | To explore nurses’ perceptions of palliative nursing in an academic hospital. | Qualitative design based on the constant comparative method Strauss. 10 nurses. Data collection – observation and interviews. Data analyses - constant comparative analysis Strauss. | Nurses adopt an organised and purposeful approach to increase well-being by acting in accordance with needs of the patient, being aware of the patient’s experiences and being available and truly present. Nurses have a caring attitude based on authentic relationships. Focus on the treatment of symptoms, observing and finding solutions. Nurses underrate the value of a caring but exhibit a caring attitude. Nurse work within the limits of ward and hospital policy and work towards finding solutions, co-ordinating care and providing information to physicians about patients. There can be an emotional stress or demand on nurse. |
| [49] Byrne and McMurray (1997) Caring for the dying: Nurses experience in hospice care.  Australia | To explore hospice nurses experience of caring for dying patients. | Qualitative phenomenological interpretative design. 9 nurses. Data collection – interviews. Data analyses – followed Colaizzi’s framework. | Nurses focused on providing care, helping patients/families and enabling patient/family to develop coping skills. Nurses develop a realistic perspective of death and dying, construct a personal philosophy, balance guilt and compassion and experiencing loss and acceptance. Nurses focus on teamwork to provide holistic care to patient/family. Conflict can occur in managing care. Caring delivered in an empathic manner that recognises one’s limitations, identifying with patients, honest open communication, intuitive listening, empowering patients to make choices, caring for family, ease conflicts, and caring for self by keeping work in perspective, managing stress and emotional distancing. |
| [50] Taylor et al (1997) Palliative nurses' perceptions of the nature and effects of their work.  Australia | To examine the nature and effects of palliative care for nurses, patients, and families. | Qualitative storytelling design. 5 nurses. Data collection -interviews. Data analyses - thematic analysis. | Nurses make connections with patients and their families, making realistic contracts and demonstrating advocacy through communicating with others. Nurse builds interpersonal relationships with family and colleague through taking time, developing trust and rapport, through bonding and getting close. Nurses support their colleagues and focus on finding solutions, facilitate breakthroughs, acceptance, and support. Nurses gain trust and facilitate engagement in care. Not always possible to make a positive difference in patient care. |
| [40] McClement and Degner (1995) Expert nursing behaviors in care of the dying adult in the intensive care unit.  Canada | To identify expert nursing behaviours in care of the dying adult in the intensive care unit. | Qualitative, descriptive, exploratory design. 10 nurses (intensive care). Data collection – interviews. Data analyses - constant comparative content analysis. | Responding to the family, providing information and reducing the potential for future regret. Providing comfort. Facilitating the transition from cure to palliative care. Responding to anger, responding to colleagues. Supporting (responding to) family after the patient has dies. Enhancing personal growth. Spending time is a prerequisite. |
| [41] McWilliam et al (1993) The challenging experience of palliative care support-team nursing.  Canada | To explore the experience of palliative care nursing as part of a multidisciplinary support team. | Qualitative phenomenological design. 2 nurses. Data collection – recorded refection and interviews, 32 reflections recorded. Data analyses - qualitative analysis unspecified. | Providing supportive care to patient and family. Valued quality-of-life and motivation to help others. Enabling the person to come to terms with what is going to happen. Facilitating other healthcare professionals. Connecting with patients, doing for, empowering, finding meaning within their work. Supporting homecare professional and coordinating and managing difficult situations. Dealing with conflict, having to adapt one’s role and preserve own integrity. |
| [42] Davies and Oberle (1990)  Dimensions of the supportive role of the nurse in palliative care.  Canada | To describe the clinical component of a supportive care nurses’ role. | Qualitative descriptive design. 1 nurse reporting on 10 cases. Data collection -interviews 25 hours. Data analyses – constant comparative analysis Glaser and Strauss. | A model of the supportive role in palliative care developed, comprised of six interwoven but discrete dimensions: Valuing, Connecting, Empowering, Doing for, Finding meaning, and Preserving own integrity. The nurse as a professional cannot be separated from the nurse as a person. The concept of support is complex. Valuing can be seen as global and having respect for the inherent worth of others through developing a deeper understanding of patients’ unique characteristics or abilities. Connecting is where the nurse spends time, finds a common bond, and establishes rapport with the patient. Empowering enables the patient/family to do for themselves by facilitating, encouraging, defusing, mending, and giving information. Doing for focuses on physical care, managing symptoms, making arrangements, lending a hand, team playing and negotiating the system. Finding meaning focuses on living and acknowledging death helping the patient to live until they die. Preserving own integrity is the ability to maintain feelings of self-worth, self-esteem and maintaining energy levels and is integral to effective functioning. |
| [43] Heslin and Bramwell (1989) The supportive role of the staff nurse in the hospital palliative care situation.  Canada | To identify supportive nursing interventions in a primary nursing palliative care hospital setting. | Qualitative exploratory design. 5 nurses. Data collection – interviews. Data analyses - qualitative analysis unspecified. | Providing holistic care, family care, personalised care (personhood), symptom control, and life closure are interconnectedness. Facilitating expression of emotions, enabling conflict resolution, and teaching effective communication strategies. Understanding the individual strengths, needs, desire so that supportive interventions of comfort, activity, rest, nutrition can be provided. Symptom control, pain control and prevent suffering. Life closure through enabling a legacy, facilitating ways to say goodbye, spirituality and pre-and-post bereavement care. |
| [44] Gotay et al (1985) Palliative home care nursing: Nurses’ perceptions of roles and stress.  Canada | To examine the ways in which a group of palliative home care nurses perceive their role and stress. | Qualitative exploratory design. 10 nurses. Data collection – interviews. Data analyses – not specified. | Palliative care nursing is a distinctive kind of nursing and nursing the terminally ill requires capabilities and provides its own unique frustrations and satisfactions. Skills included nursing skills, communication skills, empathy, flexibility, maturity, decision-making and providing support. While nurses experienced stress, they had a variety of ways to cope with stress such as talking to others, relaxing and unwinding. |
